# Particle Therapy: Clinical Applications and Biological Effects

**DOI:** 10.3390/life12122071

**Published:** 2022-12-09

**Authors:** Viktoriia Kiseleva, Konstantin Gordon, Polina Vishnyakova, Elena Gantsova, Andrey Elchaninov, Timur Fatkhudinov

**Affiliations:** 1 National Medical Research Center for Obstetrics, Gynecology and Perinatology Named after Academician V.I. Kulakov of Ministry of Healthcare of Russian Federation, 117198 Moscow, Russia; 2 Research Institute of Molecular and Cellular Medicine, Peoples’ Friendship University of Russia (RUDN University), 117198 Moscow, Russia; 3 A. Tsyb Medical Radiological Research Center, 249031 Obninsk, Russia; 4 A.P. Avtsyn Research Institute of Human Morphology of Federal State Budgetary Scientific Institution “Petrovsky National Research Centre of Surgery”, 117418 Moscow, Russia

**Keywords:** particle therapy, hadron therapy, protons, carbons, neutrons, genes, clinical applications

## Abstract

Particle therapy is a developing area of radiotherapy, mostly involving the use of protons, neutrons and carbon ions for cancer treatment. The reduction of side effects on healthy tissues in the peritumoral area is an important advantage of particle therapy. In this review, we analyze state-of-the-art particle therapy, as compared to conventional photon therapy, to identify clinical benefits and specify the mechanisms of action on tumor cells. Systematization of published data on particle therapy confirms its successful application in a wide range of cancers and reveals a variety of biological effects which manifest at the molecular level and produce the particle therapy-specific molecular signatures. Given the rapid progress in the field, the use of particle therapy holds great promise for the near future.

## 1. Introduction

Hadron particle therapy (‘particle therapy’) is an external beam irradiation cancer therapy using neutrons, protons or heavy ions. Contrary to conventional X-rays, most particle beams deliver radiation in a precise manner due to their unique physical characteristics, which minimize the collateral damage to normal tissues. This feature is particularly relevant for tumors located near critical structures and not spreading to other parts of the body. The use of particle therapy alleviates the damage to the nearby normal tissues, thus reducing the long-term side effects of radiation therapy [1].

The benefits of hadron therapy were first explored by Robert Wilson [2]. Since then, the interest in it has fluctuated significantly and is currently at its peak in developed countries. Fast neutrons, the first known hadrons, were applied clinically soon after their discovery in 1932. The first proton cyclotron for patient treatment was installed in Berkeley, USA, in 1954. The first carbon ion (C-ion) therapy center was launched at the National Institute of Radiological Science in Japan in 1994. Carbon therapy, along with proton irradiation, is a dynamically developing technology; although only 13 C-ion therapy centers are currently functioning worldwide, there are other six C-ion therapy centers under construction. The total number of particle facilities in Europe will be increased to 45 centers by the next year, 2023 [3]. In the new millennium, hadron therapy is a major focus [4].

Similar to precise drug therapy, particle irradiation requires a monitoring of the tumor and surrounding tissue responses at the molecular level. The aim of this review article is to summarize recent investigations on the clinical use of particle therapy in cancer patients and its key biological effects in order to elucidate the current state of the field and its prospects. The article is divided into two topical parts: (1) clinical applications of particle therapy and (2) ‘molecular’ responses of the tumors after particle irradiation. The first part is further divided into three sections corresponding to neutron, proton and carbon ions clinical applications. The second part includes a single section on molecular mechanisms with a focus on in vitro and in vivo models.

### 1.1. Clinical Applications of Particle Therapy

#### 1.1.1. Neutrons

Hadron radiation therapy is often regarded as a choice between protons and C-ions [5,6], whereas fast neutrons are all but neglected. Meanwhile, fast neutrons, used as radiation therapy per se or boron neutron capture therapy (BNCT), have an important advantage over X-ray irradiation—namely, oxygen independence; the curative effect results from the high linear energy transfer (LET) of fast neutrons and direct DNA damage [7]. The unique biological properties of neutron beams circumvent certain mechanisms of radioresistance [8,9]. The advantage of BNCT, which uses boron-containing drugs to deliver ^10^B to tumors, is the predominant confinement to tumors, as radionuclides tend to accumulate at the sites of tissue damage; subsequent bombardment with neutrons provides ^7^Li and an alpha particle with a short range of action [10].

Despite several remarkable clinical achievements in the 1930s, the growing evidence of severe complications in the follow-up put an end to the clinical use of fast neutrons. The approach regained some interest in the 1970s, after the extensive radiobiological research on LET, but was largely abandoned in later years.

Regardless of the widespread clinical use of photon irradiation and the growing number of proton and C-ion centers, fast neutron therapy will still represent a better option for certain tumors in particular [11]. Fast neutrons have shown undeniable advantages in the treatment of inoperable/recurrent salivary gland malignancies resistant to standard low-LET radiotherapies. A US-based retrospective cohort study evaluated 6- and 10-year survival rates and oral complication frequencies in patients receiving neutron radiotherapy for salivary gland malignancies. The 6- and 10-year survival rates for the neutron method exceeded those achieved with conventional photon irradiation at similar rates of osteoradionecrosis. Similar benefits of fast neutrons observed for other tumors were accompanied by severe toxicity. To date, there is only one functioning fast neutron center, at the University of Washington, Seattle, USA [12].

Although the severe toxic effects of using external neutron therapy limit the scope of its application, BNCT provides a fundamentally different principle of selective irradiation, delivering a large dose of radiation in 1–2 sessions with a precise focus on the cancerous lesions.

To date, the results of a phase 2 clinical trial using BNCT (JHN002) in 21 patients with recurrent head-and-neck squamous cell carcinoma or recurrent/locally advanced head-and-neck non-squamous cell carcinoma are available. Adverse side effects of the treatment included alopecia, hyperamylasemia and nausea. The two-year overall survival for the first and second groups of patients was 58% and 100%, respectively [13].

Another small prospective study carried out in Taiwan advocates for the use of two-fraction BNCT after conventional radiotherapy for recurrent head-and-neck tumors. Severe toxic effects (laryngeal edema, carotid hemorrhage) in one patient only and a survival rate of 47% among recipients allowed the authors to suggest the overall effectiveness of the two-fraction BNCT in the treatment of recurrent head-and-neck tumors [14].

The same team proposed a combination of conventional irradiation and single-fraction BNCT for this type of tumor, given the high incidence of recurrence in long-term follow-up. Apparently, in voluminous tumors, an uneven distribution of the boron formulation without the additional boost of photon irradiation may lead to incomplete elimination of the lesions. The improvement in local tumor control was confirmed in a pilot study. However, the authors abstain from proposing the use of combined therapy for patients with head-and-neck tumors of large volume on a routine basis, since a higher total dose of radiation can lead to higher toxicity. Therefore, the decision to use combination therapy is to be made on a personalized basis [15].

Another condition potentially curable with BNCT is glioblastoma (high-grade glioma, GBM), a prevalent and aggressive brain tumor. A large cohort of patients with brain tumors including malignant meningiomas and high-grade gliomas receiving BNCT in 2002–2014 (*n* = 167) was reported by Miyatake et al. [16]. Epithermal neutrons were delivered in a 12.0 Gy equivalent dose. The median overall survival time post-BNCT was 10.8 months for recurrent GBM and 15.6 months for newly diagnosed GBM. The adverse events included radiation necrosis and symptomatic pseudoprogression.

Still, reliable clinical implementation of combination and mono BNCT therapies will require more trials, given the small cohort size and tumor stage/size/localization heterogeneity in the data available to date.

In general, neutron therapy shows high efficiency in the treatment of recurrent voluminous tumors of complex localization; however, the use of fast neutron therapy is limited by high toxicity. A less-toxic variant of BNCT is still technically complex and poorly understood, therefore, it is being used as an experimental treatment available only in major academic research centers.

#### 1.1.2. Protons

Proton therapy is of great clinical interest. This would render the use of high doses of radiation a viable option. Proton therapy is considered one of the most sparing irradiation techniques, improving outcomes by means of reduced toxicity. The pronounced peak of ionizing radiation, also known as Bragg peak, appearing at the end of the charged particle’s path through the matter affords a more favorable dose distribution in non-cancerous tissues compared to photon therapy and accordingly reduces the degree of radiation-induced side effects.

Various national guidelines recommend proton therapy for 40 different types of cancer [17,18,19].

Several preclinical findings indicate that, apart from its known physical and dosimetric advantages, proton irradiation has a favorable profile of biological responses in comparison to photons [20,21,22,23,24,25,26].

To date, protons have been successfully used in pediatric cancers and adult cancers of various localization including head-and-neck, brain, lung, liver, breast and prostate tumors.

Pediatric patients are considered to benefit most from the advantages of particle therapy, since they are particularly vulnerable to the toxic side effects of irradiation, especially from the developmental perspective. The ethical difficulties of conducting full-fledged randomized trials in pediatric populations, as well as the heterogeneous characteristics of pediatric cohorts (for example, variable sensitivity to radiotherapy), determine the paucity of literature data on the long-term effects of proton therapy for this category. Still, the contribution of proton therapy to the treatment of medulloblastoma, ependymoma, craniopharyngioma, rhabdomyosarcoma and chondroma in pediatric patients is huge [27,28,29,30,31,32,33]. The use of proton therapy (craniospinal irradiation to a total dose of 36 Gy in 20 sessions) to treat medulloblastoma in 3–12-year-olds (*n* = 10) revealed a lower risk of the radiation-induced second malignancy compared to the standard photon therapy [30,34].

Similar benefits of proton therapy in medulloblastoma and supratentorial primitive neuroectodermal tumors, as applied following first-line chemotherapy, were noted in patients aged under 5 years (*n* = 15). In this study, 3-year disease-free survival was about 85%. The encountered long-term side effects included ototoxicity and endocrinopathy (two patients needed hearing aids and three patients had to start hormone therapy) [28].

With a median follow-up of 3.2 years, 3-year local control, progression-free survival, and overall survival were 85%, 76%, and 90%, respectively, for a cohort of 179 children with grade II/III non-metastatic intracranial ependymoma using proton therapy. At the same time, severe toxicity to the brainstem developed in only one patient. The authors noted the effects of gender on disease control [32].

High rates of 5-year survival (over 90%) were demonstrated in the treatment of non-metastatic chondroma in 29 patients with a median age of 14.8 years. Passive scattered proton therapy was used. It is worth noting that among the severe toxic effects developed were hormone deficiency, Eustachian tube dysfunction—which caused chronic otitis media, as well as hardware failure or associated infection [31].

In adult practice, the efficacy and safety of proton beam therapy were evaluated by Ares et al. [35]. In 1998–2005 the authors used it in patients with skull-base chordomas (historically, the first proton-treated neoplasms) and chondrosarcomas (*n* = 64). Five-year disease-specific and overall survival rates were 62% and 81% for chordomas and 91% and 100% for chondrosarcomas, respectively. Adverse toxic effects in the form of unilateral optic neuropathy and central nervous system necrosis were developed by two patients.

In a retrospective analysis of 60 patients with histologically-proven sacral chordoma at a median follow-up of 48 months, local recurrence occurred in 20 (33%) patients. The 4-year local control, no distant recurrence, and overall survival rates were 77%, 89%, and 85%, respectively. Proton therapy was used for treatment in 50 patients, and 10 patients received combined photon radiation therapy and proton therapy. Grade 3 acute toxicity developed in 11% of cases and secondary bladder malignancies in two patients. The authors conclude with the safety and efficacy of pencil beam scanning proton therapy [36]. On the other hand, a meta-analysis conducted by Iman El Sayed et al. [37] on the efficacy and toxicity of proton and photon adjuvant radiation therapy (RT) in people with biopsy-proven chordomas showed that the existing published data confirming the greater effectiveness of proton therapy have a low degree of reliability. The authors pointed to the need for prospective multi-institutional studies.

The use of proton therapy for the early stage of non-small cell lung cancer showed 3-year disease-free survival rates of up to 80% in several independent studies, and one retrospective study revealed similar results for both proton and carbon ion therapies [38,39,40].

For the locally advanced non-small cell lung cancer, characterized by high mortality and therapy resistance, proton therapy resulted in a lower incidence of pulmonary, esophageal and hematological toxicity compared to photon therapy [41]. A retrospective study by Higgins et al. [42] surveying the National Cancer Database on non-small cell lung cancer outcomes showed better survival after proton beam therapy compared with photon therapy. A study by Wong et al. [43] compared the proton and photon therapy outcomes in pediatric patients with lung metastases scheduled for whole-lung irradiation (*n* = 5) and advocated the proton therapy as a preferable, cardiac- and breast-sparing option.

Prostate cancer may be another promising area for the application of proton therapy. The clinical use of protons for prostate cancer complies with safety and efficacy requirements [44,45,46].

Another disease in which proton therapy is used is primary and metastatic hepatocellular cancer. A group from Japan achieved satisfactory results with three different irradiation protocols. The study included 266 patients, 104, 95 and 60 of whom received treatment according to protocols A, B and C, respectively; the remaining seven patients, presenting with dual-lesions, received a combination of two protocols. The 1-, 3- and 5-year overall survival rates were 87%, 61% and 48%, respectively, and the median survival was 4.2 years [47]. Bush et al. [48] published the results of a randomized trial comparing proton therapy and transarterial chemoembolization for hepatocellular cancer. The authors encountered no significant differences between the groups and the 2-year overall survival was 59%. A retrospective study by Parzen et al. [49] described individuals who received proton therapy as a treatment for hepatocellular carcinoma and intrahepatic cholangiocarcinoma (*n* = 63), with the 1-year overall survival constituting 65.6% and 81.8%, respectively.

Proton therapy is also being used to treat breast cancer. A positive clinical outcome (relapse-free 3-year period and post-radiation dermatitis not exceeding stage III) was achieved in a patient with sternal metastasis developed after conventional radiotherapy [50]. Various dose selection approaches are being tested to optimize the outcomes. Rana et al. [51] conducted a ten-patient clinical study to evaluate radiobiological and dosimetric exposure using two different irradiation protocols. The authors identified no significant differences impacting the risks of complications in normal tissues (the heart, the lungs and the skin), although one of the protocols involved a heavier dose of radiation.

A comparative study of the proton and photon irradiation effects in the treatment of breast cancer on the risks of recurrence, as well as collateral damage to the heart and the esophagus, was carried out by Raptis et al. [52]. In this study, which enrolled 12 patients, the organs at risk received significantly lower radiation doses under proton therapy [52].

Paganetti et al. [53] analyzed dosimetric data of 34 patients who received three different irradiation protocols, including protons and photons, to assess the risks of secondary malignancy in the follow-up. The results indicate reduced risks of secondary thyroid and esophageal cancers with standard photon therapy and minimal risks of lung and breast cancer with proton therapy [53]. Chung et al. [54] compared different methods of proton delivery in retroperitoneal sarcoma (*n* = 10) to observe high treatment efficacy in all cases.

The above data show that proton therapy can be used to treat malignant neoplasms that differ in their occurrence and localization. Due to the unique distribution of radiation doses specific to proton therapy, it has the following advantages over X-ray therapy: there are no harmful effects on normal tissues distal to the Bragg peak, and there is a pronounced reduction of the integral dose [55]. This is particularly important for younger patients because it is associated with a lower risk of secondary cancer, as has been demonstrated in the treatment of medulloblastoma [56] and lymphoma [57].

Proton therapy is also the method of choice for adult patients with some specific tumor types. For example, in hepatocellular carcinoma, the therapeutic effect can be achieved at a dose that, in the case of X-ray treatment, causes damage to normal liver tissue [58]. A retrospective comparative study of proton and photon ablation radiation therapy included 133 patients (median age 68 years) with hepatocellular carcinoma. The use of proton radiation therapy was shown to be associated with a higher overall survival rate and a lower risk of non-classical, radiation-induced liver disease [59]. The safety of proton therapy has also been shown in a phase 3 randomized clinical trial [60]. In the case of breast cancer, proton therapy may be more sparing for the lungs and heart [61].

Despite the significant advantages of proton therapy, there are also limitations. The main limitation is the difficulty in defining the distal edge of the proton beam when delineating the target of irradiation [55]. This is particularly complicated by changes in the patient’s anatomical parameters during treatment. In this regard, the absence of side effects and the success of therapy are largely determined by the skill of the physician. It is supposed that such limitations of proton therapy can be eliminated by using CT or MRI imaging techniques, which, in the example of hepatocellular carcinoma, allow physicians to determine the level of ascetic fluid or other features of the patient’s anatomy. Organ mobility during the treatment procedure can be another problem of proton therapy application. However, in some cases, breath holding, abdominal compression, etc. may be used to limit the mobility of the organs [55].

For many years, protons, such as fast neutrons, were only available at academic research centers. Despite being regarded as a promising option due to certain physical advantages, their use was supported by sparse clinical evidence. Over the last decade, the growing number of proton centers finally converted the prospects into reality, with new, strong evidence indicating not just the dosimetry benefits of protons, but also their underestimated biological advantages.

#### 1.1.3. Carbon Ions

The therapy with C-ions also provides several unique physical and radiobiological advantages, e.g., high conformity along with high relative biologic effectiveness (RBE) and LET. Similar to the protons, C-ions exhibit a characteristic energy distribution in depth (Bragg peak). As a consequence, distal tissues receive a small portion of energy, although, in contrast to protons, some energy is deposited distally due to nuclear fragmentation [62]. C-ion therapy has a higher LET than most other methods of irradiation as heavier ions do have higher LET. With higher LET and the specific characteristics of Bragg peak, C-ions provide a promising choice for providing higher doses to targets while reducing the collateral damage to non-target organs.

Due to the presence of Bragg peak, the ions precisely irradiate the tumor and give off energy there, while sparing the surrounding healthy tissues as much as possible and causing very moderate side effects of minimal severity. This advantage is particularly important for tumors adjacent to radiation-sensitive tissues (e.g., at the skull base, near the optic nerve or close to the intestines). The treatment accuracy is complemented by the opportunity to visualize the irradiation process and to perform real-time dosimetry, provided by the formation of unstable gamma-emitting isotopes [63]. Ion-induced ultrasound, MRI, Positron Emission Tomography and Interaction Vertex Imaging are used for imaging based on secondary radiation generation. In 2003, Stichelbaut and Jongen proposed the use of rapid gamma-ray detection (PG), and evidence for the use of this principle was later published [64,65].

C-ion therapy is a major research focus worldwide. In addition to the established protocols, more than 30 clinical studies have been registered on its use in the treatment of adult and pediatric malignant neoplasms of various localization [66], and the official page of the Particle Therapy Group [67] gives a comprehensive picture of modern centers focused on this type of radiation therapy.

A clinical study using combined irradiation of inoperable osteosarcoma with protons and C-ions was carried out in Germany. It included patients with primary, metastatic or recurrent inoperable pelvic or craniofacial osteosarcoma (*n* = 20). The 2-year overall survival reached 68% and no cases of high toxicity were observed [68].

In another study carried out in Germany, C-ions irradiation was used to treat locally recurrent pancreatic cancer after primary resection (*n* = 19). Overall survival, local control and toxicity rates were assessed 18 months after the completion of radiation therapy. The overall survival rates were comparable to those after photon therapy and the lack of difference between the two approaches was attributed to specific features of pancreatic cancer progression [69].

The use of carbon ions in colorectal cancer with metastases to the lungs and liver, attempted in Japan, also showed favorable clinical outcomes [70].

A retrospective study of clinical outcomes for oropharyngeal non-squamous cell carcinoma treated with C-ions also showed high overall survival rates and low toxicity [71].

Thus, the comparative effectiveness of conventional radiation therapy vs. C-ions is controversial, apparently due to the biological diversity of tumors.

Despite the controversy, C-ion options remain highly relevant. The nosological emphasis is placed on head-and-neck tumors and other radioresistant cancers in relapse [72,73]. Despite the sound theoretical grounds for the use of C-ions, corresponding preclinical and clinical evidence is still insufficient. The preliminary confirming data include comparative assessments of rotational volume-modulated irradiation with C-ions for the treatment of recurrent head-and-neck tumors. The use of C-ions afforded a significant reduction in the organ-at-risk dose across all patients (−8.7% D_mean_), with the dose-volume benefits most pronounced in the brainstem (−20.7% D_max_) and optic chiasm (−13.0% D_max_) [74]. C-ion effectiveness against radioresistant tumors, exemplified by photon-resistant head-and-neck cancers, has been demonstrated [75]. A promising modification of C-ion therapy is ultra-high dose rate (FLASH) irradiation, which is expected to reduce radiotoxicity. Pilot experiments using a mouse model of osteosarcoma and human fibroblasts support this assumption [76,77].

Thus, the leading field of application for C-ions will most probably be the treatment of recurrent cancers, notably those resistant to other types of irradiation. However, further research in this direction is currently needed, primarily regarding the biological aspects of the treatment. All mentioned clinical findings are summarized in Table 1.

In summary, various protocols for particle-assisted tumor therapy can provide a desirable option under certain clinical circumstances (high risks of recurrence, complex anatomical localization, low radiosensitivity, etc.). However, despite the promising preliminary results, a number of factors prevent the wider implementation of such options into clinical practice. Firstly, the safety and efficacy data for the currently available protocols of particle therapy have been obtained from small cohorts and need verification. Secondly, the choice between the options is often missing for logistical reasons (which also partly explains the small scale of clinical research data available so far). Thirdly, some tumors are virtually inaccessible without significant risks to nearby organs and their safe irradiation would require sophisticated solutions in terms of topographic anatomy. To a certain extent, the use of particle therapy is impeded by the lack of advanced molecular understanding of the biological effects of particular types of radiation.

## 2. Molecular Responses to Particle Therapy

The universal principle of any radiation therapy—the destruction of as many transformed cells as possible by directly damaging DNA—is well known since the beginning of the clinical use of irradiation. The encountered non-DNA-targeted effects of radiation can be classified as indirect effects on cell nucleus (triggered outside the nucleus and/or involving other compartments), effects transmitted from hit to non-hit cells (the “bystander” effects), delayed effects that are only expressed after a number of cell generations; and cooperative responses in which interactions between hit cells influence the overall impact. Although such effects may influence clonogenic survival, apoptosis, chromosome/chromatid damage, gene induction, genomic instability, adaptive responses and delayed lethality, they are certainly still DNA damage-dependent [81]. Despite the likely contribution of such effects, in this article we neglect them as minor and focus on tumor DNA as the major irradiation target.

The effects of particle exposure on the cell depend on multiple factors, including the dose, its microscopic distribution and spatial fractionation, as well as cell doubling time. The sparsely and densely ionizing radiation therapies can be referred to as low-LET and high-LET, respectively, and the incidence of lethal events in cells under direct irradiation positively correlates with LET value. High-LET beams destroy cells by causing direct DNA damage and almost independently of oxygen levels—their RBE is negligibly affected by the oxygen enhancement ratio, typically low in the hypoxic tumor microenvironments [82].

Moreover, high-LET particles have low selectivity with regard to the cell cycle [83] as a consequence of clustered DNA damage produced by single particles.

Rigorous research on dynamic molecular signatures in tumors is unfeasible, but the information is required for a comprehensive understanding of intrinsic mechanisms leading to either the elimination of tumor cells or the evolution of resistance. Studies in this area may provide good help both in finding ways to protect surrounding normal tissues and to kill damaged cells using modified irradiation techniques in the presence of labeling agents. The most accessible and simple research objects are in vitro models that allow the evaluation of radiation therapy’s effects on cell lines.

A number of principal molecules participating in metastatic processes, including cell adhesion molecules and integrin family proteins, were identified in a human colorectal cancer line exposed to proton irradiation [84]. Other studies identified reduced expression levels of genes involved in proliferation and repair processes, e.g., DNA-dependent protein kinase (DNA-PK), Nibrin (NBS1), Poly (ADP-ribose) polymerase (Rad51) and Poly (ADP-ribose) polymerase (PARP), as markers of metastasis [85]. Nibrin and Poly (ADP-ribose) polymerase are actively expressed upon the introduction of double-stranded breaks in DNA and can be regarded as candidate markers of successful therapy. On the other hand, upregulation of DNA repair genes, notably B lymphoma Mo-MLV insertion region 1 homolog (BMI1), alpha-thalassemia/mental retardation, X-linked (ATXR) and ATM Serine/Threonine Kinase (ATM) in response to the treatment, demonstrated in several studies [86,87], probably reflects the mechanism of radioresistance formation. Modeling of DNA damage response can be used for new prognostic tools and future therapies as a means to intervene with cancer progression and metastasis [88].

Another type of signature related to the regulation of cell cycles and reflecting the early G2/M arrest was observed in tumors after beam exposure. Such signatures encompass upregulation of genes involved in cell cycle control, notably CDKN1A, NPAT, CENPE, NEK2 and CDK1 [86,87] and downregulation of NEXN, CDC20, CDC25 and CCNA in cancer cell lines [89]. One study [87] features ATR pathway, which is activated in tumor cells, as a pivotal G2/M arrest regulator.

Yet another type of signature relates to inflammation and intercellular signaling. These encompass the increased expression of soluble pro-inflammatory factors IL-6, IL-8 and MCP-1 [90]. Other markers upregulated in response to proton therapy are p53, CDK1, and p21 (CDKN1A) ATM, IL7R, selenoprotein, GABA receptor, epsin, stefin and metallothioneins, whereas CDC25 and cyclin B2 are inhibited [86,91]. Comparisons of changes occurring under the influence of C-ions and X-rays on the immune profile showed a stronger immune response to C-ions than X-rays due to decreased secretion of immunosuppressive factors IL-10 and TGF-β.

In vitro models are useful not only in terms of understanding the mechanisms of radiation-induced tissue damage; they can also be used to select the type and mode of irradiation and adjust the conditions. For example, Suetens et al. compared the effects of C-ions and X-rays on prostate and colon cancer cell lines (PC3 and Caco-2, respectively). Significant dose- and time-dependent changes in CCDC88A, FN1, MYH9 and ROCK1 expression were revealed in both cell lines by RT-qPCR analysis. However, PC3 showed an enhanced response to C-ions, whereas Caco-2 cells responded stronger to X-rays, exemplifying tumor-specific responses to different types of irradiation [92].

Importantly, the radiation-induced molecular signatures are tumor cell line-specific, which should be considered in the clinical perspective. The accumulating experimental evidence may greatly reinforce the development of new treatment protocols with reduced side effects.

In vivo models are no less important for studying the effects of proton, neutron and C-ion beams on tumors of different origin. The widespread use of murine models to study the effects of proton therapy at a molecular level has crystallized into SIRMIO (Small Animal Proton Irradiator for Research in Molecular Image-guided Radiation-Oncology) commercial project [93]. Evaluation of particle beam irradiation parameters in mouse models helps to estimate effective doses, survival rates and tissue responses for future clinical applications [94,95]. To visualize molecular processes in tumors after proton irradiation, researchers used a patient-derived xenograft model of glioblastoma [96], medulloblastoma [97], breast carcinoma [89] and other cancers. The in vivo activated molecular signatures include CAM-driven cancer cell communication, graft-versus-host disease (due to the xenograft nature of the model) and upregulation of pro-inflammatory biomarkers [89]. Another important point is the ability of proton beams to activate a certain tumor counter-attack strategy for radioresistance through inflammation and stem cell activation, even at low doses. Increased expression levels of CD24, CD44 and CD133 were observed in breast cancer xenografts after proton irradiation [89]. Xenografts of medulloblastoma, SHH-activated, from a heavily pretreated patient who had received chemo- and radiotherapy, were implanted in the murine cerebellum. After implantation, the tumor was examined by MRI and treated with C-ion irradiation for 5 days. The treatment significantly delayed the tumor growth (median survival being 96 days in treated animals in 43 days in controls), thus providing a substantial survival benefit [97]. C-ions also prove superior to gamma irradiation when assessed in murine models [98,99]. In murine models of osteosarcoma, C-ions showed promising results in combination with immune checkpoint blockade treatment using anti-PD-1 and anti-CTLA-4 antibodies [75,100]. In addition, C-ions used in ultra-high dose rate (FLASH) mode significantly reduced lung metastasis compared to the conventional dose rate irradiation and sham-irradiated animals [77]. Neutron irradiation has been also tested in mice; for instance, Jing et al. demonstrated the dose-dependent anti-tumor effect of fast neutron beams in a pre-clinical model of cervical cancer. High doses of fast neutrons slowed down the tumor growth four-fold compared to the control group [101]. Another study assessed the positive dynamics of gene expression profiles in murine blood leukocytes following fast neutron exposure [102]. Nevertheless, neutron irradiation, which shows intersecting molecular response signatures with X-rays, is almost abandoned clinically, in contrast to proton and C-ion therapies—which are considered undeniably promising.

It is known that the effects of different types of ionizing radiation on living cells are based on similar molecular mechanisms. However, proton and neutron irradiation have different characteristics compared to X-rays [103].

A comparison of the frequency of occurrence of single- and double-chain breaks showed that more breaks are formed during the early phase of exposure with proton irradiation compared to X-ray irradiation [55].

However, this effect strongly depends on the characteristics of proton radiation [104]. The predominant repair pathway for double-stranded breaks after proton exposure is homologous recombination [105]. Complex DNA damage formation is another type of DNA damage characteristic of proton radiation compared to photon radiation, but there is little direct evidence for this [104]. As the researchers suggest, G2/M arrest is one of the specific features of proton effects on cells [106]. Another characteristic feature of proton exposure is a faster and higher increase in ROS compared to X-rays [107].

Neutrons are affected by similar mechanisms, but this type of radiation is more effective against tumors resistant to X-rays [108]. Despite this, the level of double-stranded DNA breaks under the influence of neutrons is lower than after exposure to X-rays [109]. Despite the fact that some studies show a higher efficiency of neutron therapy compared to X-rays, the specific mechanisms of this phenomenon remain understudied [110]. This may be due to the more targeted effect of neutrons on tumor cells [111].

Thus, the study of the mechanisms and pathways that promote either tumor cell elimination or resistance enables tracing the events leading to a particular outcome in more detail at all levels, from genomic to systemic. A detailed understanding of these processes will facilitate the correction of the existing particle therapy programs and deliberately select a personalized treatment scheme for each patient.

**Figure 1:**
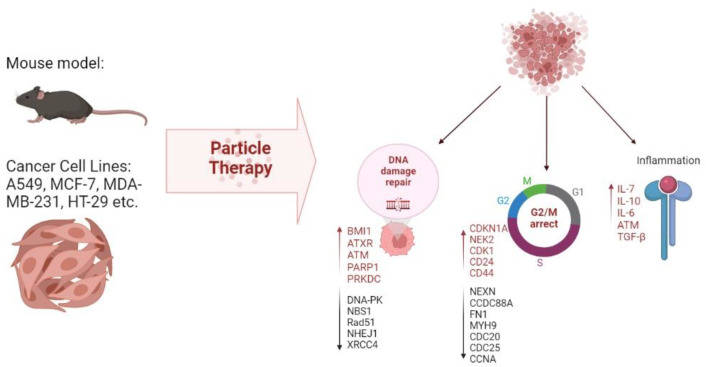
Molecular effects of particle therapy on cell lines and animal models.

All mentioned effects of particle therapy on molecular signatures are summarized in Figure 1 and Table 2.

## 3. Conclusions

Clinical examples demonstrate the great promise of particle therapy as a developing field of cancer treatment. In this review, we summarize recent advances in various types of particle therapy, encompassing both clinical findings concerning a variety of cancers and mechanistic experimental studies in vitro and in vivo. The analysis indicates that our knowledge on the mechanisms of the observed radiobiological effects, notably those of neutron therapy, is still incomplete. Understanding the correspondence between activated molecular signatures and types of irradiation will help develop personalized systems to assess the potential efficacy of the particle therapy prior to patient exposure through functional testing; for example, by using cellular extracts from tumor biopsies in order to predict the responses and minimize the adverse side effects. In addition, as already mentioned, a number of technical and organizational problems need to be solved for the widespread introduction of particle therapy into clinical practice. First, it is necessary to develop, design and establish the production of emitters convenient for a doctor and a patient; second, it is necessary to provide training of highly qualified doctors with a deeper knowledge of topographic anatomy and biophysics.

## Figures and Tables

**Table 1:** Clinical benefits of particle therapy.

Reference	Particles/Settings	Cancer Type	Number of Patients and Median Age	Study Type	Results
**Tumors of the nervous system**					
[28]	Proton irradiation	Medulloblastoma and supratentorial primitive neuroectodermal tumors	15 (35 months)	Prospective study	3-year survival ~85%
[34]	Proton and photon therapies	Medulloblastoma	8 (10 years)		Reduced risks of radiation-induced second malignancy when using proton therapy compared to conventional photon treatments
[32]	Proton therapy	Ependymoma	179 (3.2 years)	Prospective study	Confirmed high survival rate and low severe toxicity
**Cartilage and bone tumors**					
[35]	Proton therapy	Chordoma and chondrosarcoma	64 (44.5 years)	Prospective study	Confirmed safety and efficacy of spot scanning-based proton radiation therapy
[68]	Combined ion-beam radiotherapy (protons + C-ions)	Osteosarcoma	20 (20 years)	Clinical study single-center NCT01005043, completed	Positive dynamics with tolerant toxicity profile
[31]	Proton therapy	Chordoma	29 (14.8 years)	Prospective study	Low risk of serious toxicity and high efficacy
[36]	Proton therapy	Sacral chordoma	60 (59 years)	Retrospective study	Safety and efficacy of pencil beam scanning proton therapy
**Lung tumors**					
[41]	Proton and photon therapies	Lung tumors	3D Conformal Concurrent Chemoradiation (*n* = 74) (61 years), IMRT Concurrent Chemoradiation (*n* = 66) (62 years), Proton Beam Concurrent Chemoradiation (*n* = 62) (67 years)	Clinical studies NCT00495170 (completed) and NCT00991094 (continued data collection on the effects of proton therapy)	The use of high-dose proton irradiation in lung cancer has been associated with low risks of esophagitis and pneumonitis
[39]	proton therapy	Non-small cell lung cancer	56 (77 years)	Prospective study	Both protocols conferred mild toxicity
[38]	protons and C-ions	Non-small cell lung cancer	111 (76 years)	Retrospective study	Similar treatment outcomes in both groups
[40]	Proton therapy,	Non-small cell lung cancer	55 (77 years)	Retrospective study	Confirmed efficacy and good tolerability of proton therapy in non-small cell lung cancer
**Prostate cancer**					
[45]	Proton therapy	Prostate cancer	211 (68 years)	Prospective trials	High efficacy, minimal physician-assessed toxicity, and excellent patient-reported outcomes
[44]	Proton therapy	Prostate cancer	1327 (66 years)	Retrospective study	A pronounced decrease in toxicity compared with conventional methods
[46]	Proton therapy	Prostate cancer	2021 (68 years)	Retrospective study	Low toxicity and low risks of biochemical relapse
[78]	Proton therapy, photon, brachytherapy	Prostate cancer	276,880 (68 years)	Retrospective study	Proton therapy outcomes superior to photon-based external-beam irradiation and similar to brachytherapy
[79]	Proton therapy and radiation therapy	Prostate cancer	1850 (67 years)	A multicenter, retrospective study of prospectively collected data	High efficacy, low toxicity
[80]	Proton therapy	Prostate cancer	284 (64.5 years)	Prospective study	High efficacy, biochemical disease-free survival comparable with other methods, minimal risks of severe long-term toxicity
**Other cancers**					
[50]	Proton therapy	Solitary sternal metastasis of breast cancer	1 (40 years)	Case report	Complete remission at 3 years after the treatment
[54]	Proton and photon therapy	Retroperitoneal sarcoma	10 (51 years)	Comparative analysis of treatment schedules for 3D Conformal Proton Therapy, Intensity-Modulated Proton Therapy, and Intensity-Modulated Photon Therapy	High efficacy in all cases
[69]	C-ions	Locally recurrent pancreatic cancer	13 (70 years)	Prospective (clinical experience)	Overall survival rates comparable to those after photon therapy
[71]	C-ions	Oropharyngeal non-squamous cell carcinoma	33 (60 years)	Retrospective study	Improved overall survival rates and low toxicity
[70]	C-ions	Colorectal cancer with metastases to the lungs and the liver	19 (65 years)	Retrospective study	Improved clinical outcomes
[12]	Fast neutron	Salivary gland malignancies	545 (54,2 years)	Retrospective study	Improved 6- and 10-year survival rates and similar osteoradionecrosis rates compared to conventional photon radiation treatment
[72]	C-ion therapy, photon, proton radiotherapy	Sarcomas (bone and soft tissue sarcomas) and adenoid cystic carcinomas		Multicenter prospective randomized phase III trial NCT02838602 patient recruitment is ongoing on 23 March 2022.	
[74]	C-ion therapy and volumetric modulated arc therapy	head and neck cancer	16 (59 years)	Prospective study	C-ion therapy resulted in significantly reduced organ at-risk dose across all patients
[75]	C-ion therapy after photon therapy	head and neck cancer	56 (62 years)	Multicenter retrospective study	Repetitive radiotherapy using C-ions for head and neck malignancies after photon therapy is an effective treatment with tolerable toxicity.
[13]	Boron neutron capture therapy	head and neck cancer	21 (62 years)	Phase II trial JHN002 study (JapicCTI-194640)	The 2-year overall survival for recurrent squamous cell carcinoma and recurrent/locally advanced non-squamous cell carcinoma was 58% and 100%, respectively.
[59]	Proton and photon therapy	Hepatocellular carcinoma	133 (68 years)	Retrospective study	Improved overall survival rates and low toxicity
[60]	Proton and photon therapy	Hepatocellular carcinoma	144 (61 years)	Phase III trial (NCT01963429)	Safety for therapy of hepatocellular carcinoma

**Table 2:** Effects of particle therapy at molecular and cellular levels.

Reference	Experimental Model (Cell Line/Animal)	Particles	Molecular Signature
[84]	Human colorectal adenocarcinoma cell line HT-29	Protons	↓ α5β1, α6β4, αvβ3 and αvβ6 integrins; ↓ FAK and CDH1 cell adhesion molecules; ↓ RAB4, RAB11 and HAX1 integrin trafficking regulators; ↑ AMPK phosphorylation; ↑ Rab IP4 gene expression
[90]	Breast cancer cell lines MCF10A, MCF7 and MDA-MB-231	Protons	MCF10A and MCF7 cells responded dose-dependently; MDA-MB-231 cells showed strong pro-inflammatory response with ↑ IL-6, IL-8 and MCP-1
[112]	A549, MIA PaCa-2, MeWo, HuCCa-1 and U2OS cell lines	C-ions	↑ tricarboxylic acid cycle intermediates; ↑ 2-hydroxyglutaric acid oncometabolite
[89]	MDA-MB-231 cell line	Protons	↑ CD24 and CD44 gene expression; ↓ CDC20, CDC25 and CCNA gene expression; ↑ Cyclin D1; FOS activation
[113]	MCF-10A and MCF-7 cell lines	Protons	↑ DNA methylation at LINE1 elements; ↓ proliferation rate; MDH2, STYXL1, CPE, FAM91A1, and GPR37 levels significantly changed
[114]	colon tumors in CT26 mice	Protons	↑ Cxcl10 and Trex1; significant enrichment for “immune response” and “interferon signaling” GO terms identified by RNA-Seq functional profiling
[115]	A549, H520, and LLC cell lines	C-ions	↑ HMGB1; ↓ IL-10 and TGF-β immunosuppressive factors
[85]	Cal33, FaDu, HSC4, SAS, UTSCC5, UTSCC14 and UTSCC15 cell lines	Photons and protons	Similar efficacy of photons and protons shown by clonogenic survival and double-strand break repair tests
[116]	KYSE450 cell line	Photons (X-rays), protons, C-ions	All types of irradiation-induced similar immune responses regulated by STING-STAT1 axis
[97]	Medulloblastoma, SHH-activated, xenografts in mice	C-ions	↑ PARP1/PRKDC; ↓ NHEJ1, XRCC4
[89]	Breast cancer, triple-negative, xenografts in mice	Protons	↑ CD24, CD44 and CD133

↓—downregulation, ↑—upregulation.

## Data Availability

Not applicable.
